# N6-methyladenosine-dependent signalling in cancer progression and insights into cancer therapies

**DOI:** 10.1186/s13046-021-01952-4

**Published:** 2021-04-29

**Authors:** Fenghua Tan, Mengyao Zhao, Fang Xiong, Yumin Wang, Shanshan Zhang, Zhaojian Gong, Xiayu Li, Yi He, Lei Shi, Fuyan Wang, Bo Xiang, Ming Zhou, Xiaoling Li, Yong Li, Guiyuan Li, Zhaoyang Zeng, Wei Xiong, Can Guo

**Affiliations:** 1 NHC Key Laboratory of Carcinogenesis and Hunan Key Laboratory of Cancer Metabolism, Hunan Cancer Hospital and the Affiliated Cancer Hospital of Xiangya School of Medicine, Central South University, Changsha, China; 2 Key Laboratory of Carcinogenesis and Cancer Invasion of the Chinese Ministry of Education, Cancer Research Institute, Central South University, Changsha, China; 3 Department of Stomatology, Xiangya Hospital, Central South University, Changsha, China; 4 Department of Oral and Maxillofacial Surgery, the Second Xiangya Hospital, Central South University, Changsha, China; 5 Hunan Key Laboratory of Nonresolving Inflammation and Cancer, Disease Genome Research Center, The Third Xiangya Hospital, Central South University, Changsha, China; 6 Department of Medicine, Dan L Duncan Comprehensive Cancer Center, Baylor College of Medicine, Houston, TX USA

**Keywords:** N6-methyladenosine, Signal transduction pathway, Cancer progression, Therapy, RNA fate

## Abstract

The N6-methyladenosine (m6A) modification is a dynamic and reversible epigenetic modification, which is co-transcriptionally deposited by a methyltransferase complex, removed by a demethylase, and recognized by reader proteins. Mechanistically, m6A modification regulates the expression levels of mRNA and nocoding RNA by modulating the fate of modified RNA molecules, such as RNA splicing, nuclear transport, translation, and stability. Several studies have shown that m6A modification is dysregulated in the progression of multiple diseases, especially human tumors. We emphasized that the dysregulation of m6A modification affects different signal transduction pathways and involves in the biological processes underlying tumor cell proliferation, apoptosis, invasion and migration, and metabolic reprogramming, and discuss the effects on different cancer treatment.

## Background

Epitranscriptomics, a study of RNA modification and its biological functions, is a frontier field of epigenetics. Chemical modification of the RNA can induce functional epigenetic changes within the transcriptome and the modified RNA participates in various biological processes associated with post-transcriptional regulation. There are more than 160 chemical modifications of RNA [1], among which N6-methyladenosine (m6A) is considered to be the most abundant type in messenger RNAs (mRNAs) and long non-coding RNAs (lncRNAs). The m6A modification that has been reported in eukaryotes, bacteria, and viruses [2, 3] involves a methylation modification on the 6th nitrogen (N) atom of adenine (A) in RNA, with the consensus motif “RRACH” ([G > A] m6AC [U > A > C]). Similar to DNA methylation, m6A modification is also dynamic and reversible and can be co-transcriptionally deposited by the methyltransferase complex and removed by demethylase. Functionally, although m6A modification does not alter the base complementary pairing rules, it determines the distinct fate of modified RNA molecules, such as RNA splicing, transport, translation, and decay. The m6A modification of RNA plays a key role in the progression of various diseases, especially tumors. Studies have shown that m6A regulators, such as writers, erasers, and readers are often dysregulated in various types of cancer, which globally alters m6A modification abundance. Interestingly, some non-coding RNAs, including miRNA, lncRNA, circRNA, and even piRNA, have been found to change the level of m6A in cells [4–8]. Thus, accurate detection and quantification of m6A are prerequisites for a molecular-level understanding of the impact of m6A modification of RNA. Methylated RNA immunoprecipitation sequencing (MeRIP-seq), introduced in 2011, identified m6A modification abundance in humans and mice on a large-scale and high-throughput basis for the first time and revealed significant enrichment of the m6A modification in the 3′-untranslated region (3′-UTR) near the stop codon of mRNAs [9, 10]. Despite being widely used, MeRIP-seq has certain limitations, such as the need for greater amounts of RNA samples and low specificity and sensitivity. Meanwhile, some enzyme-dependent m6A modification site identification methods were introduced, such as m6A-REF-seq and MAZTER-seq, which rely on the MazF enzyme [11, 12], and DART-seq, which relies on the APOBEC1 enzyme. These antibody-free m6A modification detection methods provide new options for the precise identification of m6A modification sites. However, none of the methods identified all possible m6A modification sites. Even newly developed chemical labeling-based methods include m6A-SEAL-seq [13] and m6A-label-seq [14], which rely on the assistance of demethylase (FTO) and methyltransferases (METTL3/METTL14).

In this paper, we focused on m6A modification via regulation of the expression of different target RNA molecules affects different signal transduction pathways, which in turn, results in the regulation of the molecular mechanisms of cancer-related biological processes, including cell proliferation, apoptosis, invasion and metastasis, metabolic reprogramming, and discussed the subsequent impact of this modification on cancer treatment.

## m6A regulators: function and mechanism of action

### Writers

A multi-component m6A methyltransferase complex (MTC) that co-transcriptionally deposits m6A modification involves the heterodimer formed by METTL3/14 as a core member and WTAP, KIAA1429, RBM15/15B, and ZC3H13 as the additional auxiliary subunits. METTL3, the first discovered m6A methyltransferase, has an S-adenosylmethionine (SAM) binding domain and can catalyze the transfer of methyl groups. Importantly, when modified by SUMO1, the SUMOylation at K177/K211/K212/K215 of METL3 can significantly inhibit the activity of its m6A methyltransferase [15]. Furthermore, the phosphorylation at S43/S50/S525 of METL3 can inhibit its ubiquitination and contribute to the stability of the methyltransferase complex [16]. Although METTL14 has no catalytic activity, it can form a stable complex with METTL3 to recognize the substrate RNAs [17]. In addition, the arginine methylation of METTL14 affects the binding of METTL14 to RNA substrates and the interaction with RNA polymerase II [18]. Moreover, when the ubiquitination process of METL14 is inhibited, it contributes to the stability of METL14 and its methylation of target RNA [19]. WTAP can interact with the METTL3/14 complex and recruit it to nuclear speckles, mediating nuclear RNA m6A deposition [20]. RBM15 binds to the m6A complex and recruits it to specific RNA sites. KIAA1429 mediates 3′-UTR and m6A modification close to the stop codon by recruiting the methyl transfer complexes. It can also interact with CPSF5 to influence the length of the 3′-non-coding region of the mRNA [21]. ZC3H13 can reduce nucleation of the methyltransferase complex and improve its catalytic activity [22]. METTL16, as a newly discovered methyltransferase, catalyzes the m6A modification of hairpin (hp1) in the 3′-UTR of MAT2A. The modification induces efficient splicing, thereby regulating the homeostasis of SAM content in cells. METTL16 also binds to pre-mRNA and non-coding RNA [23, 24]. Recent findings indicate that METTL16 deposits m6A at the site of intron polyadenylation (IPA), which underscores its potential role in IPA and splicing [25].

### Erasers

FTO (alias ALKBH9), famous as an obesity-related gene, is a member of the α-ketoglutarate-dependent dioxygenase protein family and was the first m6A demethylase to be identified [26]. Subsequent studies reported that FTO can also catalyze the demethylation of m6Am at the 5’end cap of mRNA. This triggered a controversy: is the substrate of FTO m6A or m6Am [27]? Detailed research revealed that compared to m6Am, m6A is more abundant in cells, and FTO can first convert m6A into intermediate hm6A (N6-hydroxy methyladenosine), then into N6-formyladenosine (fm6A), finally becoming adenine (A) and completing the process of demethylation [28]. However, due to the structural similarity between m6A and m6Am, FTO can also act on m6Am. A reasonable explanation is that the FTO in the nucleus mainly functions in the demethylation of m6A rather than that of the end cap m6Am, while the FTO located in the cytoplasm can mediate the demethylation of both m6A and the end cap m6Am [29].

Recent studies have found that the SUMOylation on FTO will synergize with ubiquitination to cause the degradation of FTO protein, thus increasing the level of m6A in cells [30]. In addition, its deubiquitination will up-regulate FTO protein expression [31]. Another identified demethylase is ALKBH5, which is different from FTO in that it can directly remove the methyl group of m6A and directly form adenine (A) [32].

### Readers

The m6A reader protein recognizes the m6A modification and determines the fate of the modified RNA molecule, which plays a vital role in the downstream biological functions of m6A modification. Currently, there are three main types of m6A reader proteins, which contain different structural domains, have different mechanisms for recognizing the m6A motifs, and determine the distinct fate of RNA molecules. The first type of readers has a special RNA binding domain: the YTH domain (including YTHDF1, YTHDF2, YTHDF3, YTHDC1, YTHDC2, and many more), which can directly bind to the m6A motif. YTHDF1 binds to the m6A site located near the mRNA stop codon and can recruit the eukaryotic initiation factor 3 (eIF3) to promote the translation of m6A-modified mRNA. YTHDF2 recognizes the m6A modification on RNA and recruits the CCR4-NOT multi-subunit deadenylase complex by interacting with CNOT1 to accelerate the deadenylation and degradation of RNA. Interestingly, the SUMOylation of YTHDF2 can enhance its ability to bind to m6A-mRNA, thereby promoting the degradation rate of mRNA [33]. However, phosphorylation at serine39 and threonine381 of YTHDF2 has been reported to stabilize the YTHDF2 protein [34]. On the contrary, the ubiquitination modification of YTHDF2 will promote its degradation [35]. YTHDF3 can cooperate with YTHDF1 to promote protein translation and synthesis, and with YTHDF2 to promote RNA degradation [36–38]. It has been reported that all three proteins (YTHDF1/2/3) promote degradation of RNA with m6A modification [39]. However, a recent study rejected this conclusion and confirmed that m6A modification regulates the translation efficiency in various ways. This diversity partly depends on the sequence background around the m6A modification site and the binding of other RNA-binding proteins [40]. YTHDC1 is the only versatile reading protein located within the nucleus. It recruits SRSF3 while inhibiting the binding of SRSF10 to RNA, promoting alternative splicing of pre-mRNA [41]. A recent study reported that YTHDC1 induces the degradation of certain chromatin-related nuclear RNAs by interacting with the subunits of the NEXT complex [42]. A latest research reported YTHDC1 to induce the degradation of certain chromatin-related nuclear RNAs by interacting with the subunits of the NEXT complex [43]. YTHDC2 contains > 1400 amino acids. In addition to the YTH domain, it also contains various other domains with different functions. YTHDC2 can interact with MEIOC and XRN1 bridge interactions between m6A-containing mRNAs and ribosomes and improves the mRNA translation efficiency [44–46].

The m6A modification induces changes in the local RNA structure, thereby regulating the binding strength of the RNA-binding proteins to substrates. This mechanism, referred to as the “m6A-switch,” is utilized by the second type of readers to combine with m6A-containing transcripts. These readers utilize splicing factors, including HNRNPC, HNRNPG, and HNRNPA2B1, to regulate the splicing and processing of target RNA molecules, such as primary micro-RNA (pri-miRNA) and precursor mRNA (pre-mRNA) [47, 48].

**Fig. 1:**
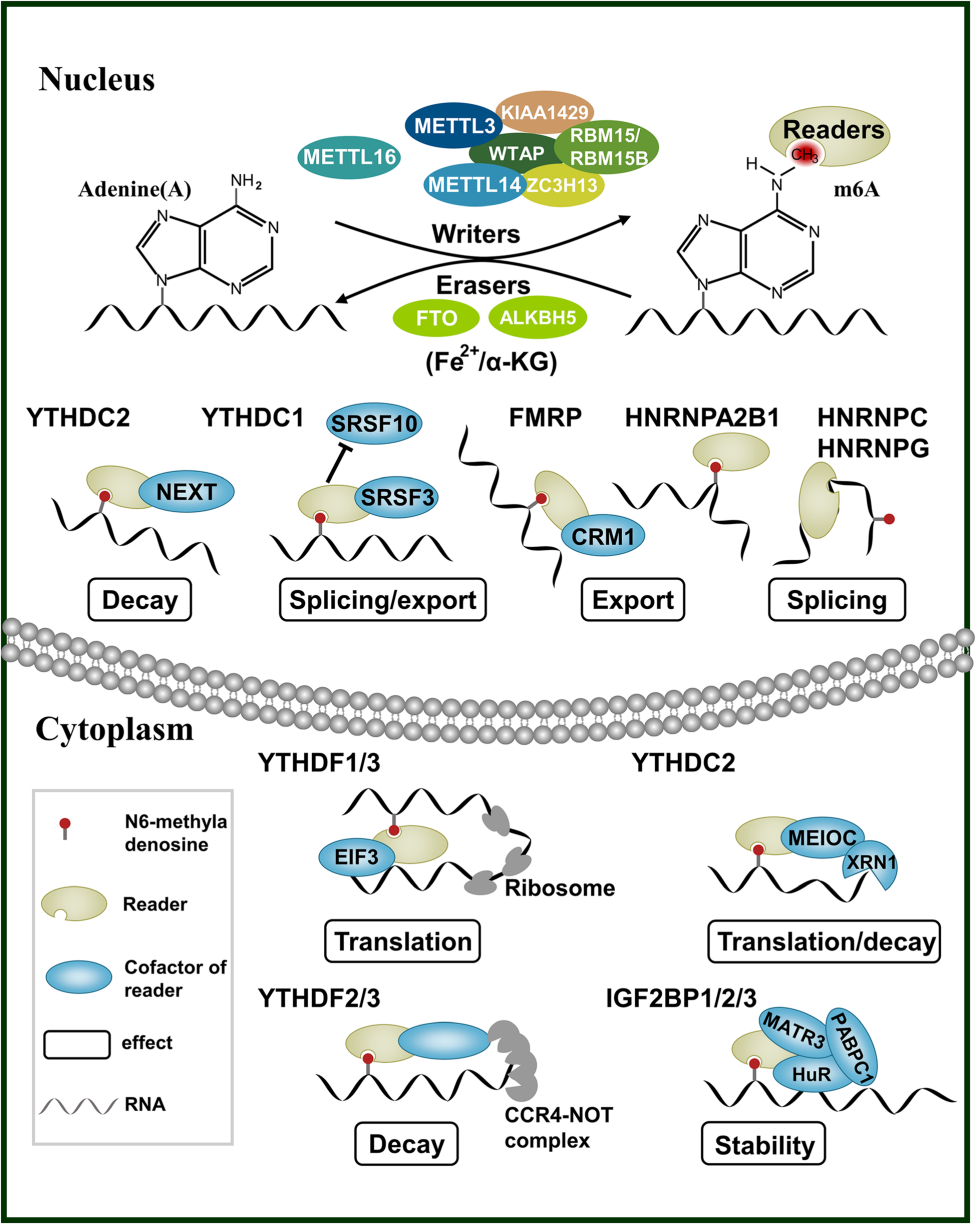
Function and mechanisms of N6-methyladenosine (m6A) regulators. The m6A modification is deposited by methyltransferase complex including METTL3, METTL14, WTAP, VIRMA, RBM15, and ZC3H13, or by METTL16 alone. The m6A modification is removed by demethylase FTO or ALKBH5. Readers could recognize and affect the fate of m6A modified RNA molecules, including RNA splicing, export, translation, decay, and so on

The newly discovered third category of reader proteins, which includes IGF2BP1, IGF2BP2, IGF2BP3, and FMRP, utilizes the shared RNA binding domains (KH structure and RGG domain) and its flanking regions to recognize m6A-containing transcripts. IGF2BPs recognize and bind to the m6A site at the 3′-UTR and enhance mRNA stability. IGF2BP2 specifically binds to HuR, MATR3, PABPC1, and other proteins, and works together with them [49]. The newly discovered reader FMRP is distributed in both the cytoplasm and nucleus, and mainly promotes the nuclear export of specific RNA [50] (Fig. 1).

## N6-methyladenosine can be associated with cancer cell proliferation

Continuous proliferation is characteristic of cancer cells [51–53]. In multiple ways m6A modification participates in cancer cell proliferation. For example, m6A modification can modulate either the regulating cell cycle processes or the various signal transduction pathways. Moreover, m6A modification can regulate the oncogenes and tumor suppressor genes at the post-transcriptional level, thereby affecting cancer cell proliferation.

Cell cycle-dependent protein kinases, CDK inhibitors, and cyclins regulate the cell cycle progression. Cell proliferation can be influenced by m6A modification of these cell cycle regulatory proteins. For example, in the G1/S checkpoint, FTO deficiency increases the level of m6A modification of cyclin D1, which is a key regulator of G1 phase progression. m6A modification promotes the degradation of the mRNA encoding cyclin D1, leading to G1/S phase arrest [54]. In addition, another study showed that m6A modification of the CDS region of CCND1 mRNA can stabilize and promote the expression of CCND1 through IGF2BP3and promote the transition of the G1/S phase [55]. Interestingly, m6A reader-cooperating lncRNA (DMDRMR) enhances the activity of IGF2BP3 and synergistically stabilizes CDK4 mRNA, leading to the G1/S transition [56]. m6A modification can stabilize CCNE1 mRNA levels and accelerate the cell cycle transition from G1 to the S phase [57]. In addition, m6A modification can promote the translation of ADAR1 mRNA through a YTHDF1-dependent mechanism, and ADAR1 plays a cancer-promoting role independently of its deaminase activity by binding CDK2 mRNA [58]. Transcript encoding E2F1, a positive regulator of the G1/S checkpoint, can be stabilized by an IGF2BP1-dependent m6A modification pathway, thus promoting G1/S cell cycle transition [59]. In the G2/M checkpoint, down-regulation of WTAP decreases the level of m6A modification, thereby blocking G2/M phase transition in liver cancer cells. Mechanistically, m6A modification promotes the degradation of ETS1 by reducing the interaction of ETS1 with the RNA stabilizing protein HUR. ETS1 can also mediate G2/M phase arrest by combining with the promoter of gene encoding p21/p27 to stimulate transcription [60]. In addition, ALKBH5 activates PER1 after transcription in an m6A/YTHDF2-dependent manner. Up-regulation of PER1 reactivates the ATM-Chk2-p53/CDC25C signal, resulting in G2/M phase transition block and inhibition of pancreatic cancer cell proliferation [61].

Modulation of the signal transduction pathways by m6A modification, changes the fate of specific RNA molecules. Signaling pathways such as AKT, nuclear factor-kB (NF-kB), Wnt/β-catenin, and mitogen-activated protein kinase (MAPK) can be regulated by m6A modification. For example, METTL3-mediated m6A modification stabilizes and promotes the expression of IKBKB, which then phosphorylates IkB activating the NF-kB signaling pathway. NF-kB dimers (p65 and p50) on entering the nucleus activate the expression of downstream MYC proteins and thereby promote cell proliferation [62]. In endometrial cancer, m6A modification promotes the translation of negative and positive AKT regulators, *PHLPP2* and *mTORC2*, in an YTHDF1-dependent and YTHDF2-dependent pathway, respectively to inhibit the AKT signaling pathway. The low expression of *METL3* and the hot spot R298P mutation in *METL14* decreases the cellular levels of mRNA encoding m6A, activates the AKT pathway, and ultimately enhances cell proliferation and tumorigenicity [63]. For example, YTHDF1 promotes the translation of key WNT receptors Frizzled5 (FZD5) and Frizzled7 (FZD7) in an M6A-dependent manner, leading to the activation of its downstream Wnt/β-catenin pathway [64, 65]. The m6A modification stabilizes and promotes the expression of *CTNNB1* encoding β-catenin, a key molecule of the classic Wnt/β-catenin pathway. High expression of *CTNNB1* activates the Wnt/β-catenin pathway [66]. The increased expression of transcription factor TCF1 by IGF2BP2-mediated m6A modification pathway, binds to β-catenin and activates the Wnt/β-catenin pathway and subsequent expression of the downstream effector molecules [67]. YTHDF2 can directly bind to and degrade epidermal growth factor receptor (*EGFR*) mRNA, inhibiting its expression. The reduced levels of EGFR inhibit the MAPK pathway, and prevent cell proliferation [68]. The METTL3-mediated m6A modification reduced the stability of *BATF2* and *RDM1* mRNA and decreased their expression. Both BATF2 and RDM1 can bind to p53 and enhance its stability, thereby inhibiting the phosphorylation of extracellular signal-regulated kinase (ERK) and subsequent ERK signaling pathway [69, 70].

m6A modification can also affect cell proliferation by regulating some oncogenes or tumor suppressor genes at the RNA level. For example, abnormal amplification of MYC-encoding MYC proteins, which are key regulators of cell proliferation, increasingly promotes proliferation of the cancer cells. In acute myeloid leukemia (AML), abnormal expression of m6A regulatory factors, including METL3, METTL14, FTO, ALKBH5, and IGF2BP1/2/3, can regulate the expression of MYC. METTL3 recruited to the chromatin by CEBPZ induces m6A modification in the coding region of target mRNA transcripts (SP1 and SP2) and promotes their translation [71]. SP1 and SP2 are important transcription factors that can bind to the promoter region of *MYC* and enhance its expression. Similar to METTL3, METTL14 regulates the m6A abundance of target mRNA (*MYB* and *MYC*), maintains mRNA stability and translation, and finally promotes the proliferation of AML cells [72]. The FTO activity inhibited by R-2-hydroxyglutaric acid (R-2HG) increases the level of m6A modification and promotes the degradation of MYC and CEBPA through an YTHDF2-dependent pathway, resulting in inhibited growth of leukemia cells [73]. Conversely, when a low level of AMPKα2 leads to an increase in the expression of FTO, the expression of MYC increases, which promotes the proliferation of colorectal cancer cells [74]. Demethylase ALKBH5 has been reported to indirectly regulate the expression of MYC. ALKBH5 directly targets and removes the m6A modification of TACC3 and promotes its expression, which then activates the expression of downstream MYC, promoting the growth of AML cells [75]. It has also been reported that m6A modification can stabilize and promote the expression of *MYC* through the IGF2BP pathway [5, 49, 76], or by stabilizing *AFF4*, maintaining the transcription of *MYC* and affecting cell proliferation [77]. Interestingly, lncRNA KB-1980E6.3 can bind to the recognition protein IGF2BP1 to synergistically stabilize c-Myc mRNA [78]. In addition to *MYC*, the well-known tumor suppressor gene *PTEN* is also regulated by m6A modification. In AML, m6A modification induced by METTL3 can promote the translation of *PTEN* [79]. In bladder cancer, METTL3 interacts with the microprocessor protein DGCR8 to promote the maturation of pri-mi*R221/222* in an m6A-dependent manner. The resultant mi*R221/222* targets and inhibits the expression of *PTEN* and promotes cell proliferation [80].

In addition to *MYC* and *PTEN*, other proliferation-related transcripts are also regulated by m6A modification. For example, the m6A modification mediated by METTL3 can maintain the stability of the transcripts of *SRSFs* [81], *HBXIP*, *ATAD2* [82], and *SOCS2* [83], promote the translation of *ANKLE1* [84], and participate in cell proliferation. On the contrary, FTO can directly target *ASB2* and *RARA* UTR, and negatively regulate the m6A level. The m6A modification reduces the stability of *ASB2* and *RARA* transcripts and promotes cell proliferation [85]. FTO can also target and remove the m6A modification of the 3-‘UTR of *BNIP3*, resulting in degradation of *BNIP3* and subsequent proliferation of the breast cancer cells [86]. The removal of m6A modification can also enhance the stability of the transcript. For example, the removal of m6A promotes the binding of *FOXM1* pre-mRNA to the RNA-binding protein HuR, and improves its stability, resulting in enhanced expression of FOXM1 protein, which in turn promotes the proliferation of the glioma cells [87]. This is consistent with the observation in renal cell carcinoma where ALKBH5 directly binds to *AURKB* mRNA in an m6A-dependent manner, enhancing the stability of the *AURKB* transcript and promoting its expression, ultimately leading to cell proliferation [88].

**Fig. 2:**
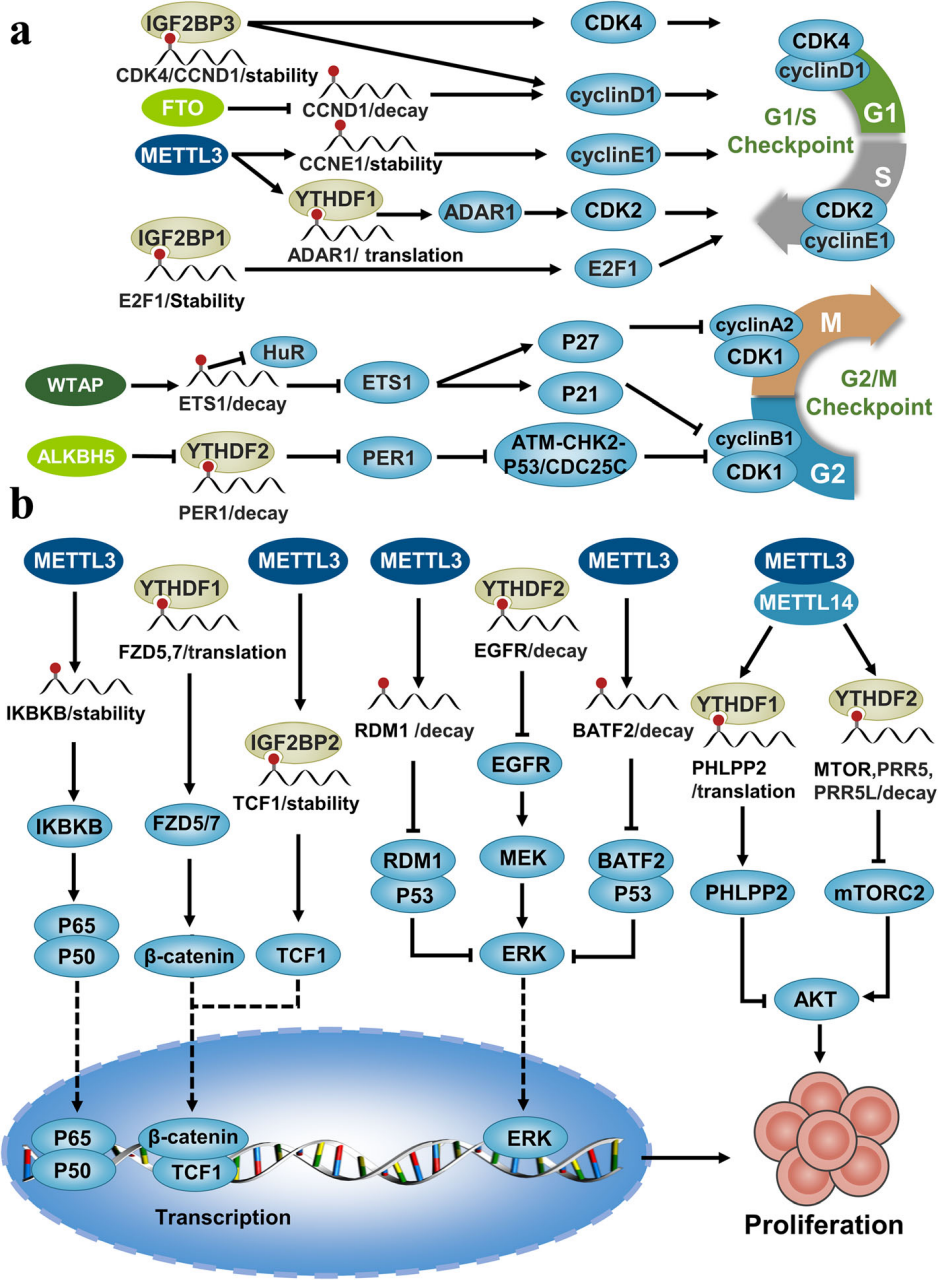
N6-methyladenosine can influence cancer cell proliferation. **a** m6A modification regulates the cell cycle and subsequent cell proliferation. At the G1/S checkpoint, m6A modification can regulate CDK4, CCND1, CCNE1, CDK2, and E2F1 post-transcriptionally, and affect the G1/S transition. While at the G2/M checkpoint, m6A modification can affect cell cycle progression by degrading ETS1 and PER1. **b** m6A modification can affect cell proliferation through affecting the fate of key molecules associated with distinct signaling pathways, such as nuclear factor-kB (NF-kB), Wnt/β-catenin, mitogen-activated protein kinase (MAPK) and AKT

The m6A modification generally affects the stability of oncogenic long non-coding RNA, which in turn regulates cell proliferation. The m6A modification alone has a positive effect on the stability of *LNCAROD*, *RHPN1-AS1*, *CCAT1*, and *CCAT2*. LNCAROD can function as a scaffold for the YBX1/HSPA1A protein complex to prevent the proteasome degradation of YBX1 [89], while RHPN1-AS1 promotes cell proliferation via miR-596/LETM1 axis [90]. CCAT1 and CCAT2 regulate the downstream MYC by enriching Let-7A and miR-145, respectively, and promote cell proliferation [91]. In addition, m6A modification can stabilize lncRNA *DANCR* through IGF2BP2-dependent pathways, and promote cell proliferation and stem cell-like properties [92]. YTHDF2-dependent m6A modification reduces the expression of lncRNA *PVT1*. On removal of m6A modification by ALKBH5, the expression of lncRNA *PVT1* and the proliferation of osteosarcoma cells were promoted [93]. In addition, m6A modification can be recognized by YTHDF1 or YTHDF2, which affects the expression of oncogenic lncRNA *THOR*, thereby affecting cell proliferation [94] (Fig. 2).

## N6-methyladenosine can influence the apoptosis-associated signaling pathway

In order to maintain the stability of the internal environment, normal cells actively and orderly trigger cell apoptosis through signal transduction mechanisms [95–101]. However, cancer cells can inhibit apoptosis by participating in these signaling pathways. According to the signal molecules that induce apoptosis, there are three main apoptosis signaling pathways: the mitochondrial, endoplasmic reticulum (ER), and death receptor pathways. The key molecules of these pathways are regulated by m6A modification to alter the apoptosis process.

For example, in the mitochondrial pathway, the Bcl-2 family of proteins regulates the apoptosis by controlling the permeability of the mitochondrial membrane. The anti-apoptotic protein Bcl-2 is located in the outer mitochondrial membrane and inhibits the release of cytochrome C. In AML, METTL3 promotes *BCL2* translation and thereby inhibits cellular apoptosis in an m6A-dependent manner [79]. In breast and ovarian cancer, the down-regulation of m6A modification level caused by *METTL3* knockout inhibits the expression of *BCL2* and accelerates cell apoptosis [102]. Moreover, in ovarian cancer, YTHDF2 can binds to BMF mRNA and promotes its degradation. BMF is a Bcl-2 family protein, which can bind to anti-apoptotic proteins, like Bcl-2, to induce cell apoptosis [35].

In the ER pathway, disruption of the ER Ca^2+^ balance or excessive accumulation of ER proteins activates the expression of Caspase 12 protein in the ER membrane and induces the transfer of cytoplasmic Caspase 7 to the ER surface. Caspase 7 activates Caspase 12 on the membrane, which then cleaves Caspase 3 to trigger cell apoptosis. SEC62, a transporter protein present in the ER membrane plays a vital role in ER pathway-mediated apoptosis. METTL3 can promote the expression of *SEC62* through m6A/IGF2BP1 pathway and thereby inhibits the apoptosis of gastric cancer cells [103].

In the death receptor pathway, the transmembrane death receptor protein on sensing external stimuli transmits the apoptotic signals through different signal transmission systems to mediate cell apoptosis. The m6A modification reduces the half-life of various m6A transcripts, including death receptor, namely tumor necrosis factor receptor *TNFRSF2* by YTHDF2. The degradation of *TNFRSF2* interrupts the TNF signaling pathway, leading to inhibition of AML cell apoptosis [104].

In breast cancer, *FTO* knockdown increases the level of m6A modification in the 3′-UTR of *BNIP3* mRNA and thereby increases its expression, which in turn induces cell apoptosis [86].

**Fig. 3:**
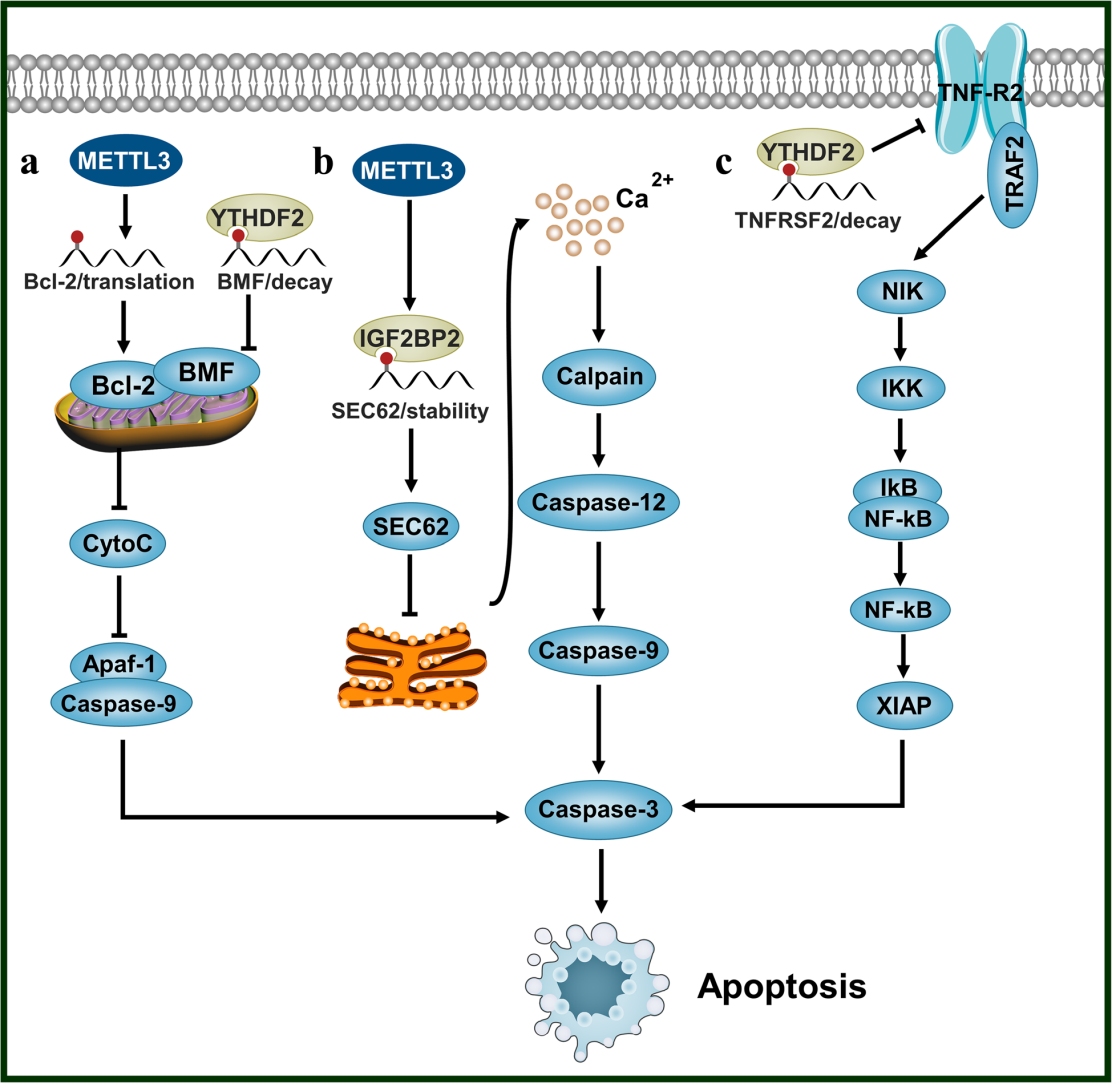
N6-methyladenosine and cell apoptosis associated signaling pathway. N6-methyladenosine can get involved in three key apoptosis associated signaling pathways as follows-. **a** In the mitochondrial pathway, m6A modification can affect cell apoptosis by regulating the fate of Bcl-2 and BMF. **b** In endoplasmic reticulum pathway, m6A modification can affect cell apoptosis by regulating SEC62. **c** In death receptor pathway, m6A modification can influences cell apoptosis by regulating TNFRSF2

In nasopharyngeal carcinoma, overexpression of *METTL3* can promote the expression level of *ZNF750*. ZNF750 can bind to the promoter of *FGF14* and promote its expression, leading to inhibition of cell apoptosis [105]. In prostate cancer, the METTL3-mediated m6A modification of *GLI1* enhances its stability and expression level. GLI1 being a transcriptional effector molecule of the hedgehog pathway, its overexpression activates the hedgehog pathway and subsequently inhibits cell apoptosis [106] (Fig. 3).

## N6-methyladenosine can regulate cancer invasion and metastasis

Invasion and metastasis are important characteristics of malignant tumors [107–113]. Regulation of the signaling pathways, such as tumor growth factor beta (TGF-β), phosphatidylinositol-3-kinase (PI3K)/AKT, MAPK, and Hippo, by m6A modification can influence the invasion and metastasis of tumor cells.

In the AKT signaling pathway, DGCR8 and the microprocessor complex are recruited by m6A modification to promote the mature processing of pri-miR-25. The miR-25 inhibits AKT negative regulator PHLPP2 and activates the oncogenic AKT-p70S6K signaling pathway to promote the progression of pancreatic cancer [114]. In colorectal cancer, *SOX4* serves as the target of METL14-mediated m6A modification. Knockdown of *METTL14* significantly reduced the m6A modification of *SOX4* mRNA, and inhibited the m6A/YTHDF2 dependent degradation pathway, thereby enhancing *SOX4* mRNA expression. SOX4 can promote the invasion and migration of colorectal cancer cells through the epithelial-mesenchymal transition (EMT) process and PI3K/AKT signaling pathway [115]. In prostate cancer, m6A degrades the tumor suppressor LHPP and NKX3–1 mRNA in a YTHDF2-dependent manner, thus promoting AKT phosphorylation and inducing tumor proliferation and migration [116]. In the MAPK signaling pathway, METTL3-mediated m6A modification can recruit the DGCR8/Drosha complex to promote the processing and maturation of pri-miR-1246. The miR-1246 inhibits SPRED2, resulting in the inhibition of the downstream RAF/MEK/ERK pathway, which in turn promotes the invasion and metastasis of colorectal cancer cells [117]. In kidney cancer, under the catalysis of extracellular adenosine triphosphate (ATP), P2RX6 can promote Ca^2+^ influx and activate p-ERK1/2/MMP9 signaling. The METTL14-mediated m6A modification promotes splicing of *P2RX6* pre-mRNA and reduces the *P2RX6* mRNA levels, thereby inhibiting invasion and migration [118]. In the Hippo pathway, METTL3-mediated m6A modification promotes the translation of *YAP* mRNA by recruiting YTHDF1/3 and eIF3b into the translation initiation complex. YAP is a key downstream effector of the Hippo signaling pathway. Overexpression of YAP promotes the invasion and metastasis of lung cancer cells [119]. Interestingly, m6A modification can also promote the expression of YAP by enhancing the stability of *MALAT1* lncRNA, which enriches miR-1914-3p through the competing endogenous RNA (ceRNA) mechanism. In addition, m6A modification can degrade lncRNA GAS5 through a YTHDF3-dependent mechanism; lncRNA GAS5 directly binds to the WW domain of YAP, promotes the translocation of YAP from the nucleus to the cytoplasm, and promotes YAP phosphorylation and YAP degradation [120].

EMT is associated with normal cell growth and homeostasis [121–123]. m6A modification regulates EMT, which in turn influences the invasion and metastasis of cancer cells. TGF-β is an important factor in inducing EMT. The m6A modification of the 5′-UTR and coding sequence (CDS) regions of TGF-β promotes the degradation of mRNA encoding TGF-β and thereby inhibits the TGF-β signaling pathway and the subsequent downstream EMT process as well [124, 125].

The demethylase ALKBH5 can remove the m6A modification of YAP, which results in its reduced expression in the non-small-cell lung carcinoma (NSCLC) through an YTHDF1/2-dependent pathway. m6A modification also inhibits YAP activity through the miR-107/LATS2 axis. This decrease in the YAP expression levels inhibits the Hippo signaling pathway and the EMT process, thereby inhibiting tumor cell invasion and metastasis [126]. In addition, some key molecules of the EMT process are also regulated by m6A modification. For example, METTL3-mediated m6A modification can promote the translation of Snail, a key transcription factor for EMT, through the YTHDF1 pathway [127, 128]. m6A modification stabilizes and promotes the expression of Snail through the IGF2BP2 pathway, and thereby affects the EMT process of cells [129]. Moreover, m6A modification can stabilize and increase the nuclear accumulation of lncRNA *RP11*, which then promotes the mRNA degradation of two E3 ligases, Siah1 and Fbxo45, by forming a complex with hnRNPA2B1. This prevents the ubiquitin-proteasome degradation of ZEB1 and promotes the EMT process of colorectal cancer [130]. Furthermore, METTL3-mediated m6A modification stabilizes and promotes the expression of ZMYM1 through a HuR-dependent pathway. ZMYM1 mediates repression of the E-cadherin promoter by recruiting the CtBP/LSD1/CoREST complex. Low expression of E-cadherin can reduce cell adhesion and promote gastric cancer cell metastasis and EMT [131]. METTL3-mediated m6A modification can degrade ZBTB4 mRNA through a YTHDF2-dependent mechanism. In addition, lower levels of ZBTB4 are associated with upregulation of EZH2, which enhances H3K27me3 combination with an E-cadherin promoter, lower E-cadherin levels, and induction of EMT [132]. m6A modification in the 3′-UTR region of *ITGA6* mRNA can promote the translation of *ITGA6* through the YTHDF1/3 pathway [133]. Moreover, m6A modification can maintain the stability of lncRNA *FAM225A*. The *FAM225A* lncRNA increases the expression of ITGB3 by adsorbing miR-590-3p/miR-1275 [134]. As a member of the integrin family, high expression of the proteins ITGA6 and ITGB3 can result in cell invasion and migration.

In addition, m6A modification can affect the RNA levels of some metastasis-related genes. For example, m6A modification stabilizes and promotes the expression of SOX2 through IGF2BP2-dependent pathways and increases the expression of its downstream targets (CCND1, MYC, and POU5F1), which in turn promotes the occurrence, invasion, and metastasis of colorectal cancer [135]. m6A modification can degrade regulators of the tumor suppressor gene, such as SETD7, KLF, and OCT4, through the YTHDF2-dependent pathway, thereby promoting cell proliferation and invasion [136, 137]. m6A modification can also promote the translation of EIF3C through the YTHDF1-dependent pathway, and at the same time enhances the overall translation output, promoting the occurrence and metastasis of ovarian cancer [138]. m6A modification can enhance the translation of ST6GALNAC5, GJA1, and EGFR mRNA through the YTHDF3 pathway and promote the brain metastasis of breast cancer cells [139]. The removal of m6A modification helps stabilize the tumor suppressors GNAO1 [30] and PERP [140], leading to inhibition of cell invasion and metastasis. ALKBH5-mediated m6A demethylation leads to post-transcriptional inhibition of *LYPD1*, which can be recognized and stabilized by IGF2BP1 [141]. Interestingly, lncRNA can assist the formation of m6A modifications. Under the guidance of GATA3-AS, KIAA1429 induces m6A modification of GATA3 pre-mRNA. The binding of HuR is inhibited by m6A modification, resulting in the degradation of GATA3 pre-mRNA, and promotes cell proliferation, invasion, and metastasis [142].

**Fig. 4:**
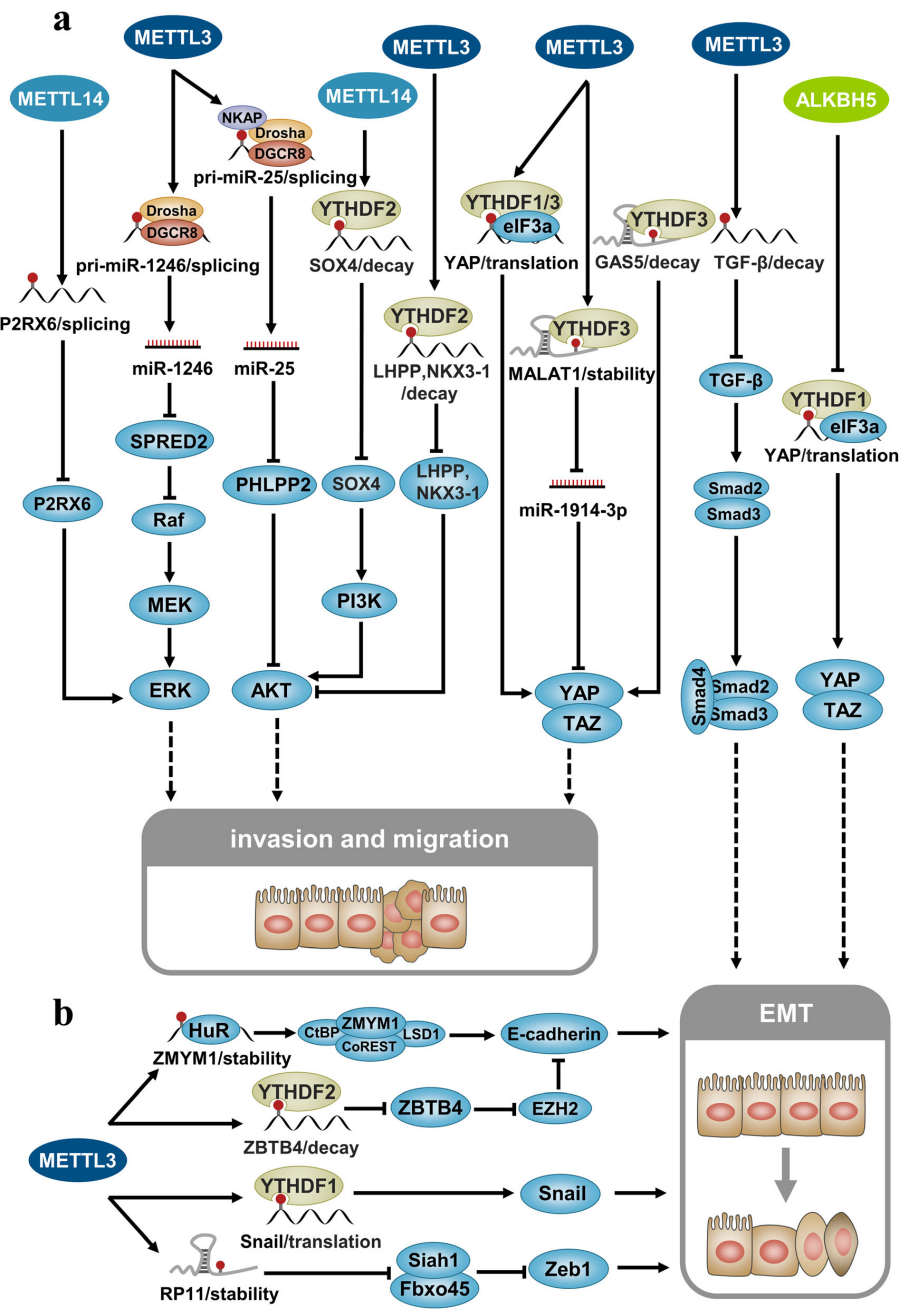
N6-methyladenosine and cancer invasion and migration. **a** m6A modification can regulate the tumor cell invasion and metastasis by affecting the different signaling pathways, including mitogen-activated protein kinase (MAPK), AKT, and Hippo signaling pathways. **b** m6A modification can affect the process of epithelial-mesenchymal transition (EMT) by regulating the tumor growth factor beta (TGF-β) and Hippo signaling pathway, or by regulating the roles of key molecules associated with EMT, such as E-cadherin, Snail, and Zeb1

It has also been reported that m6A modification can affect the fate of non-coding RNA molecules, thereby affecting tumor invasion and metastasis. For example, METTL14-mediated m6A modification recruits DGCR8 to promote the maturation of pri-miR-126 and pri-miR-375. miR-126 associated with metastasis can directly target METL14 and relieve its inhibitory effect in metastasis [143]. Conversely, miR-375 can inhibit the migration and invasion of colorectal cancer cells by targeting the SP1 pathway [144]. In addition, modification by m6A can promote the degradation of carcinogenic lncRNA XIST through the YTHDF2-dependent pathways and inhibit the proliferation and metastasis of colorectal cancer [145]. m6A modification can also stabilize LncNEAT1, which acts as a bridge between CYCLINL1 and CDK19, and promote the phosphorylation of Pol II ser2, leading to bone metastasis of prostate cancer [146]. LINC00460 directly interacts with IGF2BP2 and DHX9 to assist the recognition protein IGF2BP2 to recognize and stabilize HMGA1 mRNA, enhance the protein expression of HMGA1, and promote the proliferation and metastasis of colorectal cancer [147]. Furthermore, tandem m6A modification on the lncRNA MALAT1 can function as a scaffold to recruit YTHDC1 to nuclear speckles, thereby regulating the expression of several key oncogenes including JUN, TWIST2 and PIM1, and ultimately promoting the invasion and metastasis of cancer cells [148]. In general, the complex and variable interaction between m6A modification and non-coding RNA affects the metastasis process of cancer (Fig. 4).

## N6-methyladenosine cancer plays a role in metabolic reprogramming

Metabolic reprogramming, a characteristic of tumor cells, is essential for providing nutrients to the growing tumor [149–153]. The Warburg effect indicates that even under conditions of normal oxygen concentration, tumor cells metabolize glucose into lactate due to the expression level of the key enzymes of glycolysis. m6A modification of the glycolysis enzymes directly or indirectly regulates the expression and thereby participates in the process of tumor glycolysis.

Glucose uptake is the first step of aerobic glycolysis. METL3-mediated m6A modification stabilizes and promotes the expression of GLUT1 and increases glucose uptake directly or indirectly through the IGF2BP2/3-dependent pathways [154]. The conversion of glucose to glucose-6-phosphate depends on the enzyme hexokinase (HK). m6A modification can stabilize and promote HK2 expression through the IGF2BP2-dependent pathway [155]. The third step of aerobic glycolysis relies on the catalysis of phosphofructokinase. Studies have found that under the inhibition of R-2-hydroxyglutarate, the activity of FTO decreases, and m6A modification can degrade platelet phosphofructokinase (PFKP) and lactate dehydrogenase B (LDHB) mRNA through the YTHDF2-dependent pathway, thereby inhibiting oxygen glycolysis [156]. In addition, the expression of MYC, a regulator of glycolysis, is also enhanced by the IGF2BP2-dependent m6A modification pathway. MYC activates the transcription of related metabolic enzymes, such as GLUT1, PKM2, and LDHA [157]. Interestingly, lncRNA *LINRIS* prevents the K139 ubiquitination of IGF2BP2 and its degradation through the autophagolysosomal pathway. Knockdown of *LINRIS* inhibits the stabilizing effect of IGF2BP2 on *MYC* mRNA. PKM2 can convert phosphoenolpyruvate to pyruvate, while LDHA can convert pyruvic acid to lactate. Moreover, METTL3-mediated m6A modification can promote *HDGF* expression through the IGF2BP3-dependent pathway. HDGF in the nucleus activates the expression of *GLUT4* and *ENO2* [158]. ENO2 in turn promotes the conversion of 2-phosphoglycerate to phosphoenolpyruvate. It has also been reported that m6A modification can inhibit the aerobic respiration of cells, allowing more glucose to participate in glycolysis. For example, METTL3-mediated m6A modification can recruit eEF2 to promote the translation process of *PDK4* in an YTHDF1-dependent pathway and maintains the stability of *PDK4* mRNA through the IGF2BP3-dependent pathway. High levels of PDK4 inhibit the conversion of pyruvate into acetyl-CoA and promote aerobic glycolysis in cells [159].

**Fig. 5:**
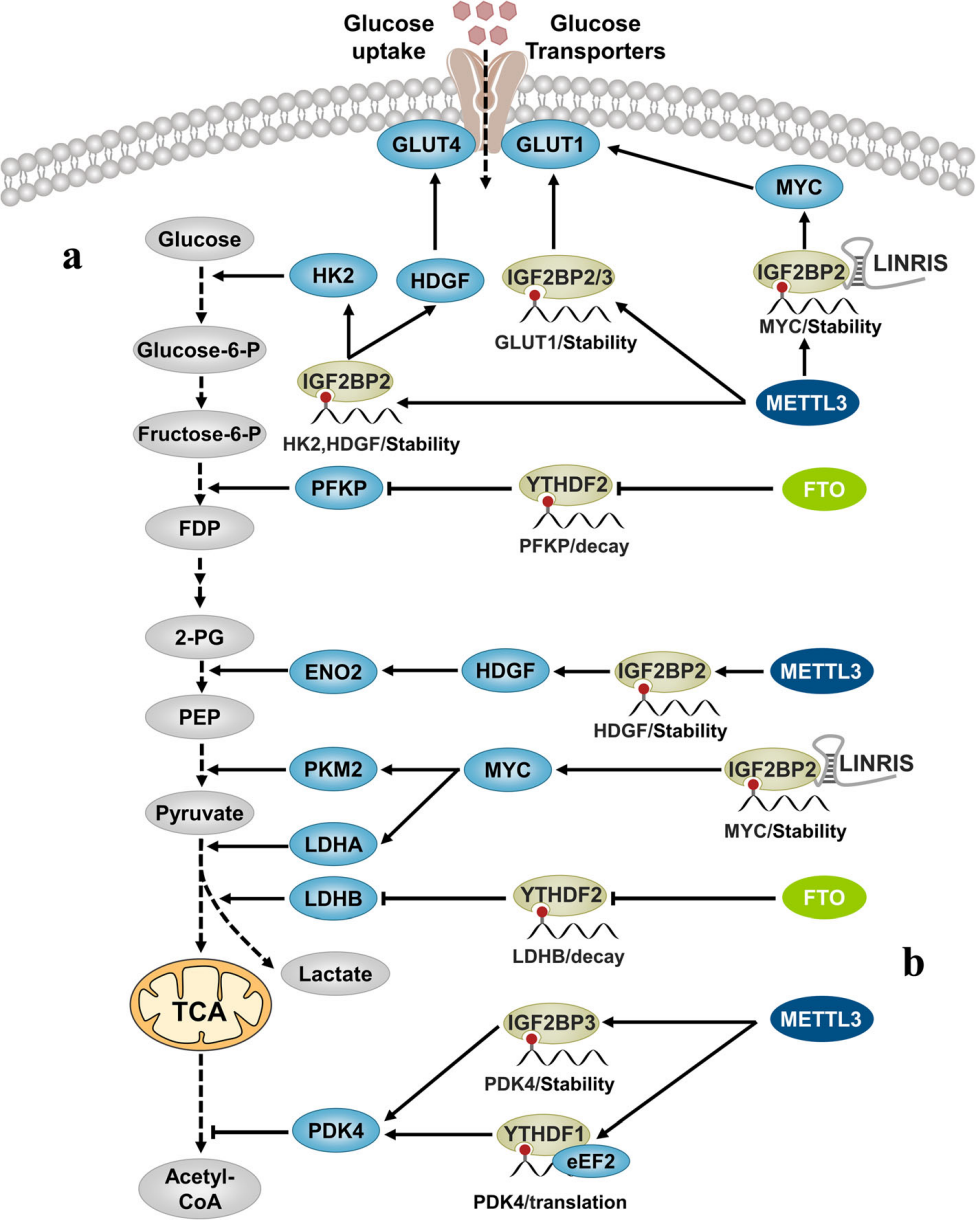
N6-methyladenosine and cancer metabolic reprogramming. **a** m6A modification can participate in the glycolysis process by affecting the RNA fate of key enzymes involved in the glycolysis process. **b** The m6A modification can also inhibit the aerobic respiration of cells, allowing more glucose to participate in glycolysis

In addition, m6A modification can stabilize and promote the expression of lncRNA *ABHD11-AS1*, which then recruits EZH2 and inhibits the transcription of *KLF2*, ultimately leading to suppression of the Warburg effect [160, 161] (Fig. 5).

## m6A modification in cancer therapies: targeted therapy, chemotherapy, radiotherapy, and immunotherapy

Many cancer studies have shown that the overall level of m6A is often dysregulated, which is caused by an abnormal decrease or increase in the m6A regulatory factors, including writers, erasers, and readers. The overall level of m6A is often dysregulated, affecting the occurrence, development, and treatment of tumors, which provides a basis for developing new cancer treatment methods. At present, some inhibitors for m6A regulatory factors that have been developed as anti-cancer agents have exhibited positive effects. Among them, the inhibitors developed against m6A demethylase FTO are the most popular. MO-I-50, the first reported FTO inhibitor, shows an ability to inhibit the survival and colony formation ability of the cancer cells in triple-negative inflammatory breast cancer cell lines [162]. Subsequently, it was discovered that a non-steroidal anti-inflammatory drug, meclofenamic acid (MA), could specifically inhibit the activity of FTO demethylase and increase the m6A level of mRNA. Further studies have confirmed that MA can inhibit the growth and survival of glioblastoma stem cells [163, 164] and enhance the efficacy of the chemotherapeutic drug temozolomide [165]. R-2HG is the main metabolite of IDH1/2 mutants. In the treatment of AML, R-2HG and DNA methyltransferase inhibitors, such as decitabine, have a synergistic effect [73]. In addition, two derivatives of MA have been found to be FTO inhibitors, including FB23 and FB23–2. Although FB23 and FB23–2 show stronger effects in inhibiting the FTO activity and viability of human AML cells, the degree of inhibition is not satisfactory [166].

Two newly reported small molecule inhibitors, CS1 (Bisantrene) and CS2 (Brequinara), can directly bind to the enzymatic reaction center of FTO, interrupting its binding with target gene mRNA and thereby inhibiting its demethylase activity. Both CS1 and CS2 compounds have broad-spectrum anticancer properties and have exhibited significant killing effects on a series of solid tumors (breast cancer, pancreatic cancer, and glioblastoma) [167]. A recent study proposed that nicotinamide adenine dinucleotide phosphate (NADP) enhances the activity of FTO, thereby regulating the level of m6A modification [168]. Furthermore, the small molecule BTYNB can disrupt the stabilization of IGF2BP1 and inhibit tumor growth [59]. In addition, the IGF1/IGF1R inhibitor Linsitinib preferentially targets YthdF2-expressing cells to inhibit GSC activity but does not affect the growth of NSCs and glioblastoma in vivo [169].

Chemotherapy, radiotherapy, targeted therapy, and immunotherapy are the common methods currently used for cancer treatment [170–178]. However, resistance of the cancer cells against these treatment options results in treatment failure and disease recurrence. The potential mechanisms for resistance to drug and radiotherapy are different, but numerous studies have revealed the potential role of m6A modification in tumor radiotherapy and drug resistance. For example, radiation can cause increased levels of m6A in lung adenocarcinoma cells, which amplifies the expression of VANGL1 through the IGF2BP2/3 pathway and activates the downstream BRAF/TP53BP1/RAD51 cascade to protect DNA from damage, thereby reducing the harmful effects of radiation on LUAD [179]. For example, FTO-mediated reduction of m6A modification can promote the expression of β-catenin and enhance the resistance of cervical squamous cell carcinoma to chemoradiation therapy [180]. High expression of ALKBH5 induces a lower m6A level and increases radioresistance of glioma stem cells by regulating homologous recombination (HR) [181]. Moreover, the reduction of m6A modification on METL3 knockout increases the sensitivity of pancreatic and glioblastoma stem cells to chemoradiation [182, 183]. m6A modification can promote the translation of IGF1R mRNA through the YTHDC2-dependent pathway, thereby activating the IGF1R-AKT/S6 signaling pathway and leading to radiotherapy resistance in nasopharyngeal carcinoma [184].

In drug resistance, the role of m6A modification can be demonstrated by the fact that it serves as an epigenetic driver of tolerance to tyrosine kinase inhibitors (TKIs). During treatment with TKI, the reduction of the overall m6A modification level mediated by FTO induces the drug-resistant phenotype of leukemia cells [185]. METTL3 can recognize the G > A mutation (R273H mutation) in the 273 codon on the pre-mRNA of *TP53* and mediate the m6A modification. This modification promotes the production of a mutant p53 protein (R273H), which then confers multidrug resistance in colon cancer cells [186]. In addition, m6A modification can promote the translation of *FOXO3* mRNA through the YTHDF1-dependent pathways, making liver cancer cells more sensitive to sorafenib [187]. However, the m6A-mediated HNF3γ reduction will cause liver cancer cells to be resistant to sorafenib [188]. Interestingly, m6A modification can stabilize CircRNA-SORE; CircRNA-SORE can act as a miRNA sponge, adsorbing miR-103a-2-5p and miR-660-3p, thereby competitively activating the Wnt/β-catenin pathway and inducing sorafenib resistance [189]. This shows that the response of m6A modification to drugs may not be single and easy to understand. In addition, the down-regulation of m6A modification can increase the sensitivity of pancreatic cancer cells to gemcitabine, 5-fluorouracil, and cisplatin [182]. Furthermore, the reduction of m6A modification can inhibit the translation of YAP through the YTHDF1/3 and eIF3b pathways, thereby enhancing the sensitivity of lung cancer cells to cisplatin [119]. Similarly, the increase of m6A modification can enhance the stability of *FZD10*, up-regulate the Wnt/β-catenin pathway, and promote PARPi resistance in BRCA-deficient epithelial ovarian cancer cells [190]. In pancreatic ductal adenocarcinoma, ALKBH5 can reduce the m6A modification on WIF1 mRNA and enhance its stability. Overexpression of WIF-1 inhibits the Wnt signaling pathway, increasing the sensitivity of pancreatic ductal adenocarcinoma cells to gemcitabine [191]. m6A modification is also closely related to cancer immune checkpoint blocking therapy. The therapeutic effect of PD-L1 blockade therapy in *YTHDF1* knockout mice is greatly enhanced [192]. In addition, FTO knockouts make melanoma cells sensitive to interferon gamma (IFN γ), thereby increasing the sensitivity of mouse melanomas to PD-1 monoclonal antibody treatment [193]. Even more exciting is that targeting m6A regulatory factors can improve the efficacy of immunotherapy. For example, deletion of METL3/14 and ALKBH5 increases the sensitivity of tumors to anti-PD-1 therapy [194, 195].

m6A modification provides a new potential option for cancer treatment. The development of new m6A editing tools may further promote the development of m6A RNA methylation research. A site-specific m6A write and erase tool has been developed to edit RNA methylation without changing the nucleotide sequence and overall m6A status [196]. The use of a dm6A clustered regularly interspaced short palindromic repeats (CRISPR) system in tumor cells to target the RNA of the oncogene *EGFR*/*MYC* can significantly reduce its expression level and inhibit the growth of tumor cells, thus revealing the potential value of the dm6A CRISPR system in cancer treatment [197].

## Conclusion

This article reviews the regulation of different cancer biological processes by m6A modification. The study elaborates on the outcomes of the different fates of modified RNA molecules on cancer treatment as a result of m6A modification. The current research initially revealed that m6A modification has a decisive effect on the fate of non-coding RNA, including microRNA, lncRNA, circRNA, rRNA, and even RNA related to chromatin regulation. Interestingly, non-coding RNA can also regulate m6A levels by regulating m6A regulators. The interaction between the two is anticipated to provide a better understanding of the role of m6A modification.

However, to accurately study the impact of m6A modification on modified RNA molecules, we first need to develop new technologies with higher accuracy, convenience, and high-throughput, as well as ones that can be applied to m6A modification imaging. Third-generation nanopore sequencing technology has been initially applied in the identification of m6A modification sites. Although false positives are high, it is expected to become more commonly used [198]. Notably, we need to determine how methylases and demethylases achieve accurate regulation of different target RNAs and figure out the link of specific m6A modification sites with the resultant phenotypes. It has been reported that miRNAs, transcription factors, histone modifications, and RNA binding proteins are involved in the specific regulation of m6A modification [199–201].

Different cancer cells have many common characteristics [202] which are targeted by the m6A modification approach through posttranscriptional regulatory mechanisms. In addition to proliferation, apoptosis, invasion and metastasis, and metabolic reprogramming, m6A modification can also interfere with the immune escape of tumor cells by regulating the presentation ability of dendritic cells and affecting the expression of immune checkpoint genes (LILRB4) [167, 192]. Studies have also initially revealed the role of m6A modification in regulating genome instability and accumulation of R-loop [203] and promoting homologous recombination repair of double-stranded DNA breaks [204].

In addition, m6A modification can also regulate the expression of *VEGFA* through the miR-143-3P/VASH1 axis [205], as well as through the regulation of *SERPINE2*, *IL11* [206], and *HDGF* stability, thus affecting tumor angiogenesis [158]. These studies have shed significant light on the research of m6A modification in cancer. Reports have revealed the profound impact of m6A modification on the treatment of tumors. Future research should focus on the development of new drugs targeting m6A modification regulators and verify their clinical efficiency to achieve effective treatment of tumors. An attractive strategy could be a combination of m6A modified target drugs with radiotherapy, chemotherapy, and immunotherapy to obtain more successful therapeutic effects.

In general, the study of m6A modification in cancer is still worth exploring. m6A modification and other epigenetic regulation, including the interaction of chromatin state, histone modification, and gene expression, are newly emerging fields of m6A modification research. Histone modifications help deliver m6A modifications to actively transcribed nascent RNA [200], and m6A modifications can also remove the methylation of the suppressive H3K9me2 corresponding to the chromatin region and activate gene transcription [207]. In addition, m6A modification can participate in the adjustment of the chromatin state by regulating the stability of chromatin-related regulatory RNAs and affecting the distribution of activated histone modifications (H3K4me3 and H3K27ac) on the chromatin. Interestingly, although YTHDF2 has been reported to induce mRNA decay, new studies have found that YTHDF2 stabilizes MYC and VEGFA transcription in an m6A-dependent manner. There will soon be more reports on the regulatory effects of m6A modification on genes at the transcriptional leve [169].

Current research has initially revealed that m6A modification has a decisive effect on the fate of non-coding RNA, including microRNA, lncRNA, circRNA, rRNA, and even RNA related to chromatin regulation. Interestingly, non-coding RNA can also regulate m6A levels by regulating m6A regulators. The interaction between the two will surely generate more sparks and help us to understand more deeply the role of m6A modification.

## Data Availability

Not applicable.

## Abbreviations

m6A: N6-methyladenosine
fm6A: N6-formyladenosine
MeRIP-seq: Methylated RNA immunoprecipitation sequencing
MTC: Methyltransferase complex
3′-UTR: 3′-untranslated region
SAM: S-adenosylmethionine
eIF3: Eukaryotic initiation factor 3
pri-miRNA: Primary micro RNA
pre-mRNA: Precursor mRNA
NF-kB: Nuclear factor-kB
MAPK: Mitogen-activated protein kinase
EGFR: Epidermal growth factor receptor
ERK: Extracellular signal-regulated kinase
AML: Acute myeloid leukemia
TGF-β: Tumor growth factor beta
EMT: Epithelial-mesenchymal transition
ceRNA: Competing endogenous RNA
CDS: Coding sequences
NSCLC: Non-small-cell lung carcinoma
